# The Epidemic Risk of Dengue Fever in Japan: Climate Change and Seasonality

**DOI:** 10.1155/2021/6699788

**Published:** 2021-10-21

**Authors:** Xia Wang, Hiroshi Nishiura

**Affiliations:** ^1^School of Mathematics and Information Science, Shaanxi Normal University, Xi'an 710062, China; ^2^Kyoto University School of Public Health, Yoshidakonoecho,Sakyoku, Kyoto 6068501, Japan

## Abstract

Dengue fever is a leading cause of illness and death in the tropics and subtropics, and the disease has become a threat to many nonendemic countries where the competent vectors such as *Aedes albopictus* and *Aedes aegypti* are abundant. The dengue epidemic in Tokyo, 2014, poses the critical importance to accurately model and predict the outbreak risk of dengue fever in nonendemic regions. Using climatological datasets and traveler volumes in Japan, where dengue was not seen for 70 years by 2014, we investigated the outbreak risk of dengue in 47 prefectures, employing the temperature-dependent basic reproduction number and a branching process model. Our results show that the effective reproduction number varies largely by season and by prefecture, and, moreover, the probability of outbreak if an untraced case is imported varies greatly with the calendar time of importation and location of destination. Combining the seasonally varying outbreak risk with time-dependent traveler volume data, the unconditional outbreak risk was calculated, illustrating different outbreak risks between southern coastal areas and northern tourist cities. As the main finding, the large travel volume with nonnegligible risk of outbreak explains the reason why a summer outbreak in Tokyo, 2014, was observed. Prefectures at high risk of future outbreak would be Tokyo again, Kanagawa or Osaka, and highly populated prefectures with large number of travelers.

## 1. Introduction 

Dengue fever is a mosquito-borne infectious disease, seen in most tropical and subtropical areas in the world. It is reported by the World Health Organization (WHO) that dengue cases have increased dramatically around the world, identifying Americas, South-East Asia, and Western Pacific regions as the most seriously affected regions [1]. In 2019, the Philippines reported 420,000 cases and Malaysia reported 131,000 suspected cases of dengue.

In Japan, dengue epidemic was continuously observed from 1942 to 1945, and, afterwards, the country remained dengue-free for about 70 years. In these days, Japan is not a dengue-endemic country, and approximately 50 to 200 imported and confirmed cases are annually reported, which is considered to be increasing steadily [2]. In 2013, the possibility of autochthonous dengue in Japan was suspected, because a German tourist returning from Japan and without any recent history to dengue-endemic countries was diagnosed as dengue fever when the patient was back in Germany. Dengue has become a threat to many nonendemic countries where the competent vectors such as *Aedes albopictus* and *Aedes aegypti* are abundant. WHO has reported that local transmission was seen for the first time in France and Croatia in 2010 [1]. Subsequently, the first local outbreak in Tokyo was also reported in 2014, involving 160 confirmed dengue cases from August to October [3]. In this way, imported cases can contribute to inducing local transmissions of dengue in Japan, indicating that any temperate zone countries that welcome travelers from endemic countries face the threat of local dengue outbreak.

Owing to an increase of the traveler volume and the importation of dengue in Japan, the investigation of the outbreak risk is increasingly recognized as more important than before. Nakamura et al. studied the risk of dengue among Japanese travelers and demonstrated that the risk is greater during epidemic season in the tropics [4]. Fukusumi et al. studied the monthly and yearly notification trends of dengue among Japanese travelers and found that the trend among Japanese travelers closely reflected the transmission dynamics in the travelers' destinations, varying seasonally and annually [2]. Yuan and Nishiura estimated that the size of infected travelers may be more than 20 times the notified number of confirmed imported cases [5]. These published studies underscore the critical importance of travelers from dengue-endemic countries in characterizing the outbreak risk in Japan.

Climatological factors have been reported to be influential to many mosquito-borne diseases including dengue fever. It can not only affect the life cycle of mosquitoes but also affect the transmission probability and incubation period. The effective reproduction number varying with time, and thus with climatological variables, has been studied by various researchers, accounting for the relationship between climatological factors and epidemiological parameters, such as the biting rate and transmission probability per bite [5–10]. Assuming that the basic reproduction number is the mean of the offspring distribution of a Galton–Watson branching process, the probability of extinction or outbreak of a disease can also be assessed [11, 12]. However, such assessment did not take place in the published studies of dengue fever, while seasonal variation of the reproduction number was dealt with. The purpose of the present study is to analyze the risk of local outbreak of dengue by prefecture in Japan, considering epidemiological impact of climatological factors and the number of imported cases on the risk of outbreak. We thereby provide fundamental insights into the time-dependent outbreak risk to which the future control strategy can be aligned.

## 2. Materials and Methods

### 2.1. Data Source

Fukusumi et al. [2] indicated that 70–90% of imported cases in Japan are from India, Indonesia, Thailand, and Philippines. Adding Malaysia to this list, the analysis of the cumulative risk from 2006 to 16 by Yuan and Nishiura [5] shows that these five countries are the five leading countries of origin of dengue for Japan. Thus, here we investigate the dataset of travelers from these five countries. Monthly datasets of travelers arriving from India, Indonesia, Philippines, Thailand, and Malaysia from 2013 to 2016 were obtained from the Japan National Tourist Organization (http://www.jnto.go.jp/jpn/statistics/visitor_trends/indexhtml). Annual dataset of travelers' destination in Japan, 2013, was also extracted from the Japan National Tourist Organization. The distribution of prefectures that were visited by travelers was obtained. Combining two pieces of data, the distribution of prefectures by country of origin and month was obtained.

The annual total numbers of imported cases from 2013 to 2016 were derived from nationwide surveillance data (National Epidemiological Surveillance of Infectious Diseases (NESID)), as shown in Table 1. The dataset enabled us to capture yearly variations of imported cases which may be caused by different epidemic dynamics in countries of origin, associated with dominant serotypes and genotypes. Subsequently, the monthly number of imported cases by prefecture was calculated by multiplying the total number of imported cases to the distribution of travelers by month and prefecture. The monthly temperature by prefecture from 2013 to 2016 was also retrieved from Japan Meteorological Agency (http://www.jma.go.jp/jma/index.html).

### 2.2. The Effective Reproduction Number

Let *μ* be the mortality rate of mosquito vector, *r* be the recovery rate of human host, *m* be the vector-to-host ratio, *a* be the biting rate of vector, *b* be the transmission coefficient from human to vector, and let *c* be the transmission coefficient from vector to human. Then, the effective reproduction number can be written as follows [13–15]:

(1) $$\begin{array}{c} R\left(T\right)=\frac{ma^{2}bc}{r\mu }e^{-\mu \text{EIP}}, \end{array}$$

where *T* is the monthly mean temperature and EIP is the extrinsic incubation period of the virus.

*Aedes albopictus* is the major vector species of dengue transmission in Japan. Of the six temperature-dependent parameters in the expression of the effective reproduction number, to our knowledge, only the mortality rate and the biting rate are available for *Aedes albopictus* as the published evidence [16, 17], and they are quantified as

(2) $$\begin{array}{c} \mu \left(T\right)=1-\max\left\{\mu_{A},0.04417+0.00217T\right\},\\ a\left(T\right)=\max\left\{-0.004981T^{2}+0.274T-2.94,0\right\}, \end{array}$$

where *μ*_*A*_=0.02 and 1−*μ*_*A*_ stands for the maximum adult mortality rate. Due to the shortage of studies on *Aedes albopictus*, known temperature-dependent relationship over the transmission probability [6, 8] and the extrinsic incubation period were derived from those for *Aedes aegypti*. Besides, a reduction factor of 0.7 was multiplied to the probability of transmission per bite to human (*c*) for *Aedes albopictus* relative to *Ae. Aegypti*, based on experimental evidence [6, 15, 18]. The relationships between temperature and the transmission probability and extrinsic incubation period of *Aedes aegypti* are described as

(3) $$\begin{array}{c} b\left(T\right)=0.001044T\left(T-12.286\right)\sqrt{32.461-T},\;12.286\leq T\leq 32.461,\\ \tilde{c}\left(T\right)=\left\{\begin{array}{c} 0.0729T-0.9037,\\ 1, \end{array}\right)\begin{array}{c} 12.24\leq T\leq 26.1,\\ 26.1\leq T\leq 32.5. \end{array} \end{array}$$

Expected length of the extrinsic incubation period duration is [15]

(4) $$\begin{array}{c} \text{EIP}\left(T\right)=4+e^{5.15-0.123T}. \end{array}$$

The infectious period of dengue is estimated to range from 4 to 12 days [1], and it is usually assumed to be 5 days [19]. The vector host ratio *m* was set to be 0.37, by fitting the default value of the reproduction number at 3 in August in Tokyo.

### 2.3. A Model for Dengue Extinction

We consider a statistical model to assess the probability of extinction of dengue in Japan. As shown in published studies [11, 12, 20], the branching process model has been employed to estimate the probability of extinction of various diseases by assuming that the basic reproduction number to be the mean of the offspring distribution. To account for the effect of climatological factors on the transmission of dengue, we assume that the reproduction number varies with temperature. We thus employ the time-varying branching process to approximate the probability of extinction of dengue over the course of calendar time.

Denote *Z*_*n*_ is the number of cases of generation *n*; *Y*_*n*,*i*_ is the number of secondary cases generated by the *i*-th case in generation *n*. If *Z*_0_=1, then

(5) $$\begin{array}{c} Z_{1}=Y_{0,1},\\ Z_{2}=Y_{1,1}+\cdots +Y_{1,z_{1}},\\ \vdots \\ Z_{n}=Y_{n-1,1}+\cdots +Y_{n-1,z_{n-1}}. \end{array}$$

The branching process is {*Z*_0_, *Z*_1_, *Z*_2_,…}={*Z*_*n*_ : *n* ∈ *N*}.

Suppose that the offspring distribution of generation *n* is a distribution with mean *R*_*n*_ where *R*_*n*_ is the effective reproduction number of generation*n*. Denote the probability generating function (p.g.f.) for *Y*_*n*,*j*_ by *G*_*n*_(*s*)=∑_*l*=0_^*∞*^*p*_*n*,*l*_*s*^*l*^, with probability *P*(*Y*_*n*,*i*_=*l*)=*p*_*n*,*l*_, and let *G*_*Z*_*n*__(*s*)=∑_*k*=0_^*∞*^*q*_*n*,*k*_*s*^*k*^ be the p.g.f. for *Z*_*n*_. The following composition is obtained:

(6) $$\begin{array}{c} G_{Z_{n}}\left(G_{n}\left(s\right)\right)=\overset{\infty }{\underset{k=0}{\sum }}q_{n,k}{\left(\overset{\infty }{\underset{k=0}{\sum }}p_{n,k}s^{l}\right)}^{k}=\overset{\infty }{\underset{k=0}{\sum }}\sum_{l_{1},l_{2},...,l_{k}}q_{n,k}p_{n,l_{1}}p_{n,l_{2}}\ldots p_{n,l_{k}}s^{l_{1}+l_{2}+\cdots +l_{k}}. \end{array}$$

One finds that this is a p.g.m. for *Y*_*n*,1_+*Y*_*n*,2_+⋯+*Y*_*n*,*Z*_*n*__ and just *Z*_*n*+1_. So, we have *G*_*Z*_*n*__(*s*)=*G*_*Z*_*n*−1__(*G*_*n*−1_(*s*)). By descending the recursion, with taking *Z*_0_=1 being encoded by *G*_*Z*_*n*__(*s*)=*G*_0_(*G*_1_(…*G*_*n*−1_(*s*))…), the mean of *Z*_*n*_ is

(7) $$\begin{array}{c} E\left(Z_{n}\right)=E\left(Z_{n-1}\right)E\left(Y_{n-1,i}\right)=E\left(Y_{0,i}\right)E\left(Y_{1,i}\right)\ldots E\left(Y_{n-1,i}\right). \end{array}$$

Let *p*_*e*_*n*__ be the probability that the process is extinct by generation *n*; that is,

(8) $$\begin{array}{c} p_{e_{n}}=P\left(Z_{n}=0\right). \end{array}$$

Then,

(9) $$\begin{array}{c} p_{e_{n}}=G_{Z_{n-1}}\left(G_{n-1}\left(0\right)\right)=G_{0}\left(G_{1}\left(\ldots G_{n-1}\left(0\right)\ldots \right)\right), \end{array}$$

because by the definition of the p.g.f., we have

(10) $$\begin{array}{c} G_{Z_{n}}\left(s\right)=P\left(Z_{n}=0\right)+P\left(Z_{n}=1\right)z^{1}+P\left(Z_{n}=2\right)z^{2}+\cdots \end{array}$$

If *Z*_0_=*n*_0_, then the extinction probability by generation *n* will be (*p*_*e*_*n*__)^*n*_0_^. So, the probability that the disease is not extinct by generation *n* can be shown as 1 − (*p*_*e*_*n*__)^*n*_0_^, which can be also denoted by the outbreak probability. If there are *I* imported cases, each imported case can be treated as the initial of a branching process. Denote *p*_*e*_*ni*__,  *i*=1,2,…, *I* to be the extinction probability of the *i*th imported case in generation *n*. Then, the total extinction probability will be *p*_*e*_*n*1__*p*_*e*_*n*2__ … *p*_*e*_*nI*__. Thus, the outbreak probability will be 1 − *p*_*e*_*n*1__*p*_*e*_*n*2__ … *p*_*e*_*nI*__.

Here, the offspring distribution of generation *n* is denoted to be a negative binomial distribution with mean *R*_*n*_ and dispersion parameter *k*, which includes the Poisson distribution (1/*k*⟶*∞*) and geometric distribution (*k*=1) as special cases. Then, the probability generating function (p.g.f.) for *Y*_*n*,*i*_ is *G*_*n*_(*s*)=1/[1+*R*_*n*_*k*(1 − *s*)]^1/*k*^. Substituting into (1), the probability of extinction if one dengue infectious individual is introduced is subsequently obtained.

## 3. Results

### 3.1. Traveler Volumes

**Figure 1:**
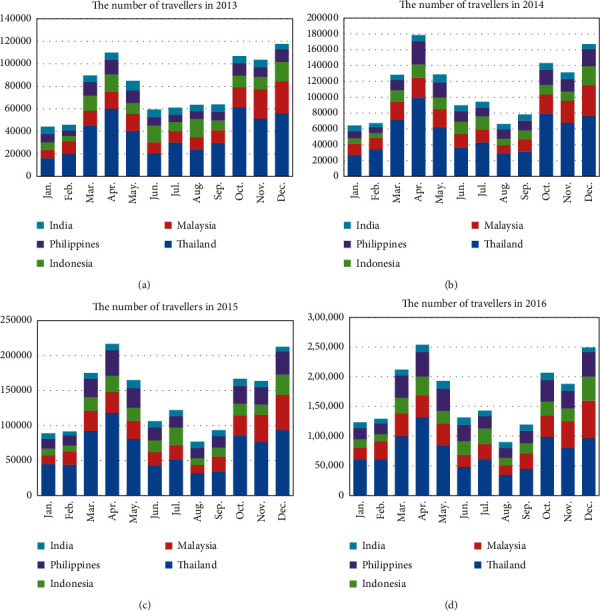
The distribution of travelers.

Figure 1 shows the distribution of travelers from India, Indonesia, Philippines, Thailand, and Malaysia from 2013 to 2016. As shown in the figure, distributions of travelers in these four years share common qualitative patterns. Travelers were dominated by visitors from Thailand and Malaysia. There are two peaks of traveler volumes in one year. One is from March to May and the other is from October to December. Comparing four subfigures, the number of travelers from 2013 to 2016 has increased with time.

**Figure 2:**
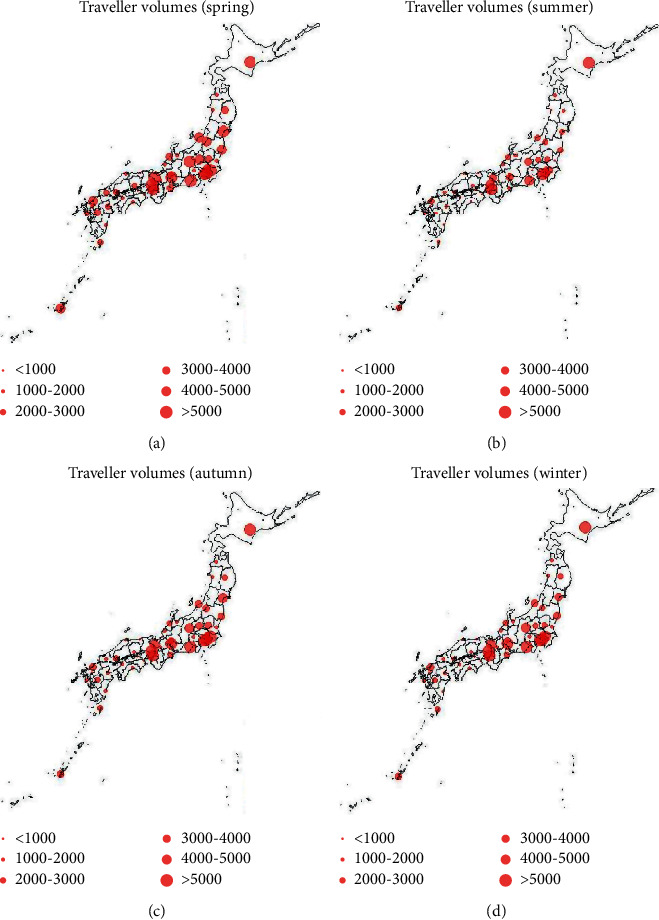
Seasonal mapping of traveler volumes. Red points represent the monthly traveler volumes of each prefecture.

To investigate the geographic distribution of the travelers by season, the map of traveler volume in 2015 was illustrated (Figure 2). According to seasonal climate conditions in Japan, one year is divided into four seasons: spring (from March to May), summer (from June to August), autumn (from September to November), and winter (from December to February). Figure 2 reveals that the traveler volume in spring is the largest and the traveler volume in summer is the lowest. Moreover, travelers' favorite place is the North and Midland of Japan, and Hokkaido, Tokyo, and Osaka are the most common destinations. The average monthly volumes of these three prefectures are higher than 5000 persons regardless of the season. Besides, Chiba, Kanagawa, Shizuoka, Kyoto, and Hyogo are also identified as prefectures with high number of travelers. The monthly number of travelers of the most prefectures in the southern Japan are smaller than 2000 persons in summer, autumn, and winter.

### 3.2. The Effective Reproduction in Tokyo

**Figure 3:**
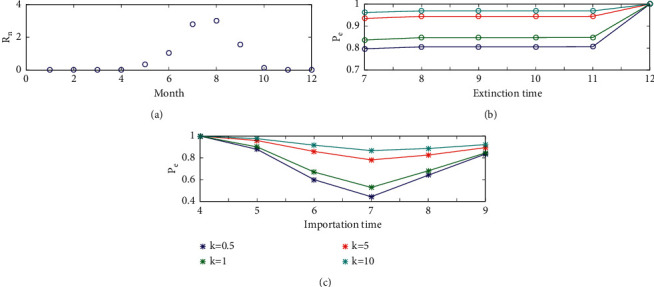
(a) Seasonality of the effective reproduction number of Tokyo. (b) The probability of extinction when the infectious individual is imported in June. (c) The probability of extinction in November versus importation time.

Substituting the monthly mean temperature in 2013–2016 into the expression of parameters in (2)–(5), we can get the monthly variation of the biting rate, probability of transmission, extrinsic incubation period and the mortality rate. Of these, monthly biting rate and mortality rate are theoretical values and computationally obtained from (2). The monthly variation of the effective reproduction number in Tokyo is shown in Figure 3(a). It follows from this figure that the effective reproduction number is almost zero in the winter season and early spring (from November to April) and reaches its peak in July and August.

The generation interval consists of four components, that is, (i) the mean extrinsic (mosquito) incubation period (5–15 days), (ii) the mean intrinsic incubation period (4–7 days), (iii) mean host infectious period (3–7 days), and (iv) mean adult mosquito lifespan (6–15 days) [21]. We assume the generation interval to be a constant 30 days, and then there would be exactly one generation per month. Let the effective reproduction number to be the same within one month. Using the expression of the extinction probability for the branching process as shown in (1), we can calculate the extinction probability if one infectious individual is imported into Tokyo.  Figure 3(b) shows the probability of extinction from July to December with different values of the dispersion parameter *k*, when an infectious individual is imported in June. This figure indicates that the probability of extinction from July to November is similar, and it finally reaches 1 in December due to the low temperature; such dependence is preserved when the parameters under multiple generations and varying temperature are being taken into consideration. Besides, large dispersion parameter leads to a big extinction probability. So, in the following, we mainly study the extinction probability in November considering different importation time.

To understand the month in which the outbreak risk is the highest, the probability of extinction in November versus different importation time, when one infectious individual is imported into Tokyo is shown in Figure 3(c). Effects of different values of dispersion parameter on the extinction probability are also shown in the figure. It indicates that the probability of extinction in November is 1 if an infectious individual is imported when the temperature is very low. If the infectious individual is imported in summer, the extinction probability would be far smaller. The lowest extinction probability reaches when the infectious individual is imported in July. Besides, the value of the dispersion parameter can affect the extinction probability greatly. When *k*=0.5, the extinction probability by November if the infectious individual is imported in July is only 0.44. However, when *k*=10, the extinction probability by November if the infectious individual is imported in July is 0.87. The figure also shows that the difference of the extinction probability among these different values of *k* is smaller when the infectious individual is imported in the month with relative low temperature.

### 3.3. The Geographical Distribution of the Effective Reproduction Number

**Figure 4:**
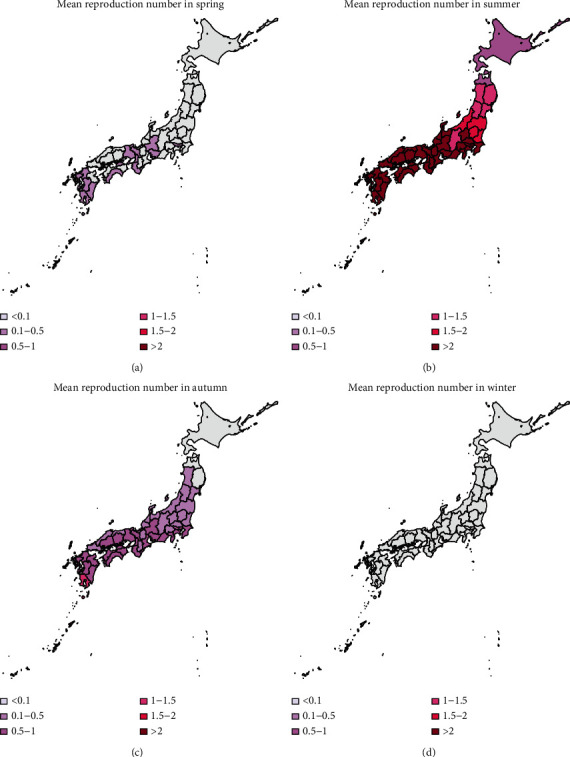
Seasonal mapping of reproduction number. Winter: December–February; spring: March–May, summer: June–August; autumn: September–November.

To compare the effective reproduction number by prefecture and season, we drew the heat map of the mean reproduction number in 2013–2016 in different seasons for the 47 prefectures as shown in Figure 4. It follows from this figure that the reproduction number in summer is much higher than that in other seasons. In summer, the effective reproduction numbers in all prefectures except for Hokkaido and Aomori are higher than 1. Besides, in autumn, only in Kagoshima and Okinawa, the effective reproduction number is higher than 1. The reproduction numbers in spring and in winter in all prefectures are lower than 1. Obviously, the reproduction number in winter is the lowest, which is below 0.1.

### 3.4. The Geographical Distribution of the Outbreak Risk

**Figure 5:**
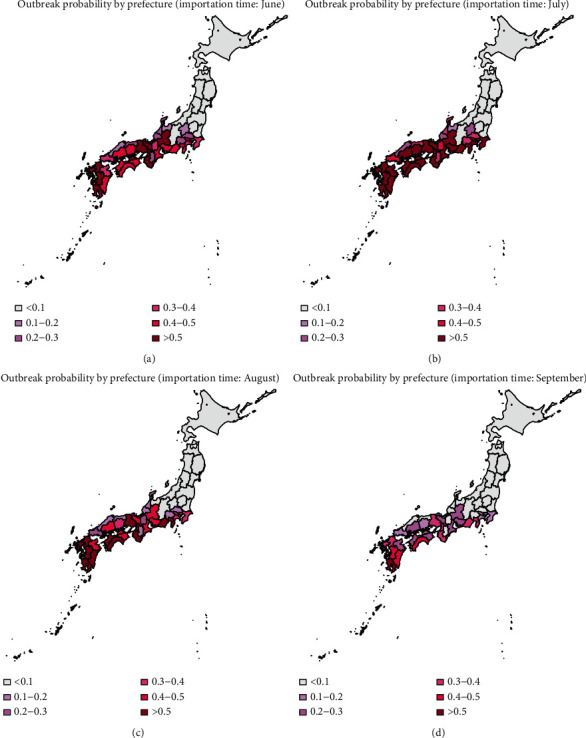
Heat map of the mean probability of outbreak in November (2013–2016), when the infectious individual is imported in June, July, August, and September; *k*=0.5.

**Figure 6:**
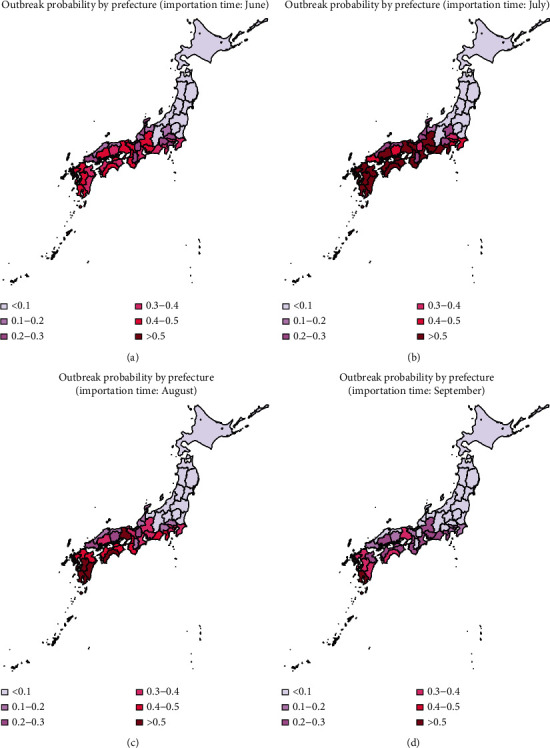
Heat map of the mean probability of outbreak in November (2013–2016), when the infectious individual is imported in June, July, August, and September; *k*=1.

**Figure 7:**
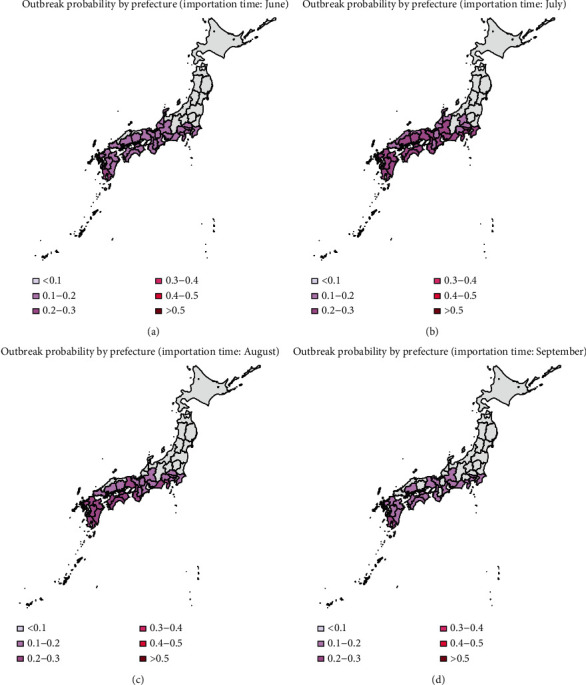
Heat map of the mean probability of outbreak in November (2013–2016), when the infectious individual is imported in June, July, August, and September; *k*=5.

The heat map of the mean outbreak probability for the 47 prefectures using data from 2013 to 2016 is shown in Figures 5–7, in which the dispersion parameter *k* = 0.5, 1, and 5, respectively. According to the results of Figure 3, the probability of extinction from July to November is similar, and it finally reaches 1 in December because of the low temperature. The outbreak risk in this study is defined as the probability that the dengue fever is not extinct until November. From Figures 5–7, we can see that importation of infectious individual in July results in the highest risk of outbreak. Besides, comparing these three figures, we can see that the outbreak risk when *k*=5 is the lowest. In other words, small dispersion parameter leads to a high probability of outbreak. When *k*=5, the outbreak risks in all prefectures are lower than 0.3 even in summer. However, when *k*=0.5, the outbreak risks in the south of Japan are much higher. We can see clearly that there is a high risk of outbreak in prefectures in the south of Japan, especially in the southern coastal areas. Miyazaki, Kagoshima, Nagasaki, and Okinawa are always the prefectures with highest risk (above 0.4). However, in northern coastal areas such as Tottori, Shimane, and Fukui, although they are also in the south of Japan, there is a lower probability of outbreak (below 0.3). Besides, it is interesting that Shiga and Nara are also with relatively low probabilities of outbreak although prefectures around them are at high risk. Shizuoka, Gifu, Aichi, and Kanagawa form the boundary of the high risk and low risk areas. All prefectures located in the north of these four prefectures have a very low probability of dengue outbreak.

**Figure 8:**
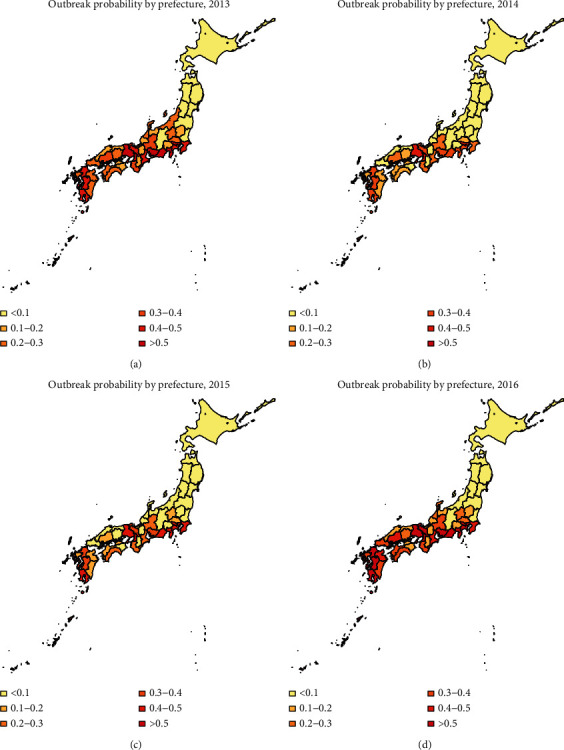
Heat map of the actual probability that dengue fever sustains until November; *k*=0.5.

In Figures 2 and 5, we have shown the traveler volume and the outbreak risk when one individual is imported. To combine the traveler volume into the outbreak risk, the actual outbreak risk is assessed in the following. According to the geographical distribution and the monthly distribution of travelers and incorporating the number of imported cases every year as shown in Table 1, we can calculate the average imported cases in each month and each prefecture. As shown in Figures 5–7, the outbreak risk is higher when *k* is smaller. We consider the case of *k*=0.5 in the following analysis. The map of the actual probability that dengue fever sustains until November can be shown in Figure 8. Comparing four figures, we identify that the outbreak risk in 2015 was the lowest. Figure 8 indicates that prefectures with the highest risk are Tokyo, Shizuoka, Osaka, Hyogo, Aichi, and Okinawa, where the outbreak risks in 2013 and 2016 were higher than 0.9. Chiba, Kanagawa, Mie, Kyoto, Fukuoka, Kumamoto, and Kagoshima were also at very high risks, those of which are larger than 0.7 in 2013 and 2016. It is interesting to observe that some prefectures in the southern Japan, including Nagano, Toyama, Fukui, Shiga, Tottori, Shimane, and Tokushima are also at low risk (<0.1 in 2014 and 2015, and <0.3 in 2013 and 2016).

## 4. Discussion

In this study, we investigated the outbreak risk of dengue fever in Japan, accounting for the traveler volume and temperature of every prefecture. First of all, according to the travel data, the distribution of traveler was obtained over month, prefecture and country of origin. Then, using the relationship between temperature and transmission parameters, the effective reproduction number was estimated by month and prefecture. To assess the outbreak risk of dengue fever, the theory of branching process was used to calculate the probability of extinction, thereby yielding the outbreak risk of dengue across all 47 prefectures in Japan.

The time distribution of travelers in Japan showed that the numbers of travelers in March, April, May, October, November, and December in 2015 are larger than 150000 persons, while the number of travelers in summer is far smaller. However, the map of the effective reproduction number indicates that the reproduction number reaches its peak in summer, which is bigger than 1 in most prefectures in Japan. Also, it is higher in autumn than in spring, which is similar to the results of the previous paper studying the relative vectorial capacity of dengue in Japan 2004–2013 [3].

In published studies, the Galton–Watson branching process has been used to estimate the basic reproduction number and the probability of extinction of some diseases, such as influenza and pneumonic plague [12, 20, 22]. The subcritical, critical, and supercritical cases can be explicitly examined. In subcritical and critical cases, the extinction probability is 1, while in supercritical cases, there is a positive probability of extinction. However, in fact, the reproduction number is time-varying due to many factors, such as climatological factors and control measures. In the present study, we used time-varying branching process to model the transmission of dengue, assuming that the offspring distribution was negative binomially distributed with mean *R*_*n*_ and dispersion parameter *k*. As shown in Figure 4, the effective reproduction number varies largely by season and by prefecture. So, the probability of extinction if one infectious individual is imported varies greatly by variations in the time (season) of importation and geographic position.

According to our results, July is at the highest risk of outbreak, and the south and central areas of Japan are high risk areas (as shown in Figure 5). However, incorporating effect of traveler volumes, the actual outbreak risks in many prefectures as shown in Figure 8 are much higher than those shown in Figure 5, especially in Chiba, Tokyo, Kanagawa, Shizuoka, Kyoto, Osaka, and Hyogo. Moreover, there are also some prefectures not at high risk as expected, such as Nagano, Toyama, Fukui, Shiga, Tottori, Shimane, and Tokushima. Although the reproduction numbers in these prefectures are also very large, the traveler volumes are not so high, leading to relatively small outbreak risks compared with urban locations. Hokkaido is a tourist attraction in Japan with more than 5000 persons per month throughout the year; it has very low outbreak risk due to low temperature. Tokyo was always the prefecture at the highest risk, not only because of high temperature but also due to large travel volumes. The finding is consistent with observing the local outbreak in Tokyo in 2014 [23–25].

Furthermore, according to the results of Figure 4.1, the traveler volume has increased over time from 2013 to 2016. Larger traveler volumes would lead to larger outbreak risk. However, as shown in Figure 4.8, the outbreak risk in 2015 is much lower than that in 2013 and 2014, due to the low temperature in 2015. Besides, it is shown in Figure 5 that tourists in June and July need special attention, and according to Figure 1, there are more tourists from Thailand and Indonesia in June and July, but the number of tourists from Thailand and Malaysia ranked the top two in the whole year. So, the number of tourists and outbreak risk are not necessarily a simple positive proportional relationship. This indicates that it is not advisable to judge the probability of outbreaks solely based on the number of imported cases or climatological factors. Multiscale model with multiple factors, especially mobility, would act as the key element of dengue prediction [26].

The variance-to-mean ratio for the negative binomial distribution is 1+(*R*_0_/*k*), so the smaller values of *k*, the greater heterogeneity of infectious individual. To study effect of heterogeneous transmission, sensitivity analysis for the dispersion parameter *k* was carried out by investigating the probability of extinction under different values of *k*. Our results indicate that greater heterogeneity of infectious individual leads to a larger probability of extinction. Also, the differences in the probability of extinction under different values of *k* is larger, if the reproduction number becomes greater. These results are consistent with the published study of individual level variations in disease transmission [20, 27, 28]. Besides, according to the actual situation of Japan, there is no local outbreak of dengue fever before 2014, so the outbreak probability should not be too large, and to give a lower limit of *k* to interpret risk maps, it is appropriate to assume *k*=0.5.

Our study provides a method to assess the outbreak risk of vector borne disease, considering both climatical factors and traveler volumes. It can predict where would be at the highest risk of outbreak and when would be the most hazardous time of importation. As the number of travelers increases, increasing the strength of surveillance targeting travelers would be required, and mosquito control may have to be intensified. Geographical distributions of outbreak risk can provide fundamental insights for public health departments into developing control plans. The proposed method could also be applicable to many other seasonally varying infectious diseases. It can allow us to consider effects of control measures on the probability of extinction. However, there are also some limitations. We assumed that the distribution of imported cases mirrored the distribution of traveler volumes. In fact, dengue fever is seasonal disease, and the number of imported cases may not only depend on the traveler volume but also depend on the prevalence in the country of origin [2, 5]. More precise estimation can be attained if the prevalence is taken into account. As an important assumption that is conventionally adopted in other published studies, the ratio of human to mosquito and the generation interval were assumed as constant. More realistic and quantitative models are called for, and the presented framework in the present study would act as the basis for such future improvement.

## Figures and Tables

**Table 1:** Numbers of imported cases.

Year	2013	2014	2015	2016
Imported cases	249	178	292	338

## Data Availability

The present study rests on mathematical modelling. Simulation results data are available upon request.
